# Supplementary material for: Region-specific laboratory reference intervals are important: A systematic review of the data from Africa
Source: PLOS Glob Public Health. 2022 Nov 14;2(11):e0000783. doi: 10.1371/journal.pgph.0000783 (PMC10021479; doi:10.1371/journal.pgph.0000783)
Supplement: Attachment — Submitted filename: Authors response to review of PGPH-D-22-00497.docx [file pgph.0000783.s003.docx]

Re: PGPH-D-22-00497
Region-specific laboratory reference intervals are important: a systematic review of the data from Africa

***Response to review:***

1. Please amend your detailed Financial Disclosure statement. This is published with the article. It must therefore be completed in full sentences and contain the exact wording you wish to be published.

supported your study, including funding received from your institution. 
- State the initials, alongside each funding source, of each author to receive each grant.

***Authors’ reply: We have revised the funding statement for clarity, please see page 12. It was previously completed in full sentences, however – just in case we are not talking about the same thing, our financial disclosure statement should read:***

***This IAVI study was entirely funded by The Bill & Melinda Gates Foundation (reference number INV-024949). No author received any funding from any other source. The funder played no role in the data collection, analysis or writing of the manuscript, or the decision to submit it for publication.***

2. Please provide separate figure files in .tif or .eps format.

***Authors’ reply: We have revised the resubmitted figures to an acceptable format.***

3. We do not publish any copyright or trademark symbols that usually accompany proprietary names, eg (R), (C), or TM  (e.g. next to drug or reagent names). Please remove all instances of trademark/copyright symbols throughout the text, including ®, TM on pages 43 and 31.

***Authors’ reply: Thank you for pointing this out. We have removed these instances.***

4. Figure 2: please (a) provide a direct link to the base layer of the map used and ensure this is also included in the figure legend; (b) provide a link to the terms of use / license information for the base layer. We cannot publish proprietary or copyrighted maps (e.g. Google Maps, Mapquest) and the terms of use for your map base layer must be compatible with our CC-BY 4.0 license.  If your map was obtained from a copyrighted source please amend the figure so that the base map used is from an openly available source. Alternatively, please provide explicit written permission from the copyright holder granting you the right to publish the material under our CC-BY 4.0 license. Please note that the following CC BY licenses are compatible with PLOS license: CC BY 4.0, CC BY 2.0 and CC BY 3.0, meanwhile such licenses as CC BY-ND 3.0 and others are not compatible due to additional restrictions.  If you are unsure whether you can use a map or not, please do reach out and we will be able to help you. …

***Authors’ reply: This is the same open-source, public domain vector map we have been using for nearly 20 years. Unfortunately, the exact provenance is unknown. We are a non-profit and have always employed open-source options for figures like this, and have used this map hundreds of times in reports, manuscripts, and the like. Kindly let us know if this is sufficient, or if not, how you would like to proceed.***

5. We have noticed that you have uploaded Supporting Information files, but you have not included a list of legends. Please add a full list of legends for your Supporting Information files after the references list.

***Authors’ reply: We have added our list of legends for the supporting information files***

Additional Editor Comments (if provided): Thank you for providing a descriptive analysis of your work. We look forward to your resubmission of a revised manuscript based on comments and suggestions from the reviewers. We look forward to receiving a revised manuscript with policymaking implications in addition to the descriptive analysis.

***Authors’ reply: Thank you for your comment. We have revised our discussion section, and provided more details on policymaking implications***

5. Review Comments to the Author

Reviewer #1: 1. The word "summary" not necessary to say please change to "Abstract "

***Authors’ reply: We have made this change.***

2. There are a lot of work "we" through out in the document please remove it

***Authors’ reply: I am not certain what you mean with this comment. If you’re talking about the use of first person plural (“we”) throughout the manuscript, we find that an active tense makes for a more enjoyable and informative reading experience. We didn’t feel this was a necessary change, but thank you for your comment.***

3. Location of abbreviation list inappropriate

***Authors’ reply: Thank you for this comment. We will work with the editors of PGPH to ensure the location of this list is more appropriate.***

4. Discussion section totally not like discussion just description of the your finding please rewrite it

***Authors’ reply: Thank you for your comment, though more detail would have been helpful. We have revisited the discussion section and hope you find it more like discussion.***

5. General the manuscript not followed this journal guidelines

***Authors’ reply: Thank you for your comment, we have now followed the journal’s guidelines (for more details, find our replies to the editors at PGPH above)***

Reviewer #2: The authors showed genuine effort in their methodology to achieve the objective of the study. They also showed results that is relevant to scientific and clinical practice. However I find the conclusion weak in recommending the application of newer and standardized methods of RI generation studies, though this was mentioned.

 ***Authors’ reply: Thank you for this feedback. As we conducted this review, we observed that while there were many studies on generating RIs to include, most suffered from very serious methodological limitations. We learned more about the IFCC efforts to conduct a global, multicenter study on reference values in a manner that allowed comparisons across regions, but highlighted that this represents a very small minority of the studies reviewed. Our conclusions are to encourage and support efforts to adhere to this “Global RI Study”, and to seek funding to facilitate this. We have revisited the discussion section with this in mind, and hope you find the new version stronger.***

Are there reasons for limiting the literature search to work done after 2006? Considering that this study is on reference intervals for analytes in clinical diagnosis and trials, less emphasis on 'focusing on recent work' may have improved robustness of findings. Kindly clarify this choice in the method section.

***Authors’ reply: We conducted one of the largest (at the time) multicenter studies on RIs in Africa, and had done a review of the literature in Africa at the time of publication of this study (2009). Our study was conducted in 2004-2006. There wasn’t much published at that time, and we were interested to review what had happened in the time since our study. We hope we have made this clearer in the methods and discussion.***

While revising your submission, please upload your figure files to the Preflight Analysis and Conversion Engine (PACE) digital diagnostic tool …. PACE helps ensure that figures meet PLOS requirements. To use PACE, you must first register as a user. Registration is free. Then, login and navigate to the UPLOAD tab, where you will find detailed instructions on how to use the tool. If you encounter any issues or have any questions when using PACE, please email PLOS at [figures@plos.org](mailto:figures@plos.org). Please note that Supporting Information files do not need this step.

***Authors’ reply: Thank you for this tip and link. We have sent our figures through PACE.***
